# Hot-Electron-Activated Peroxidase-Mimicking Activity of Ultrathin Pd Nanozymes

**DOI:** 10.1186/s11671-020-03388-9

**Published:** 2020-08-11

**Authors:** Yonghua Tang, Xueqing Xiong, Chengjie Xu, Deshuai Yu, Yanyan Huang, Changxu Lin, Xiangyang Liu, Youhui Lin

**Affiliations:** 1 Research Institute for Biomimetics and Soft Matter, Department of Physics, Fujian Provincial Key Laboratory for Soft Functional Materials Research, Jiujiang Research Institute, Xiamen University, Xiamen, 361005 China; 2 College of Light Industry and Food Engineering, Nanjing Forestry University, Nanjing, 210037 China; 3 Department of Physics, National University of Singapore, 2 Science Drive 3, Singapore, 117542 Singapore

**Keywords:** Nanozymes, Ultrathin Pd nanosheets, Peroxidase-mimicking, Visible light, Hot electron

## Abstract

Light-activated nanozymes can provide a wealth of new opportunities for the chemical industry and biotechnology. However, present remote-controlled catalytic systems are still far from satisfactory. Herein, we present an interesting example of applying ultrathin Pd nanosheets (Pd NSs) as a light-controllable peroxidase mimic. Since most of Pd atoms are exposed on their surface, Pd NSs with a thickness of 1.1 nm possess high peroxidase-like activity. More importantly, under light excitation, such intrinsic activity can be further activated by a nearly 2.4- to 3.2-fold. Such a phenomenon can be ascribed to the unique optical property of ultrathin Pd NSs, which can efficiently capture photons to generate hot electrons via surface plasmon resonance effect and thus promote the in situ decomposition of H_2_O_2_ into reactive oxygen species radicals (O*). This enhanced catalysis can also be used for real-time and highly sensitive colorimetric detection of H2O2. We expect our work can provide valuable insights into the rational design of artificial nanozymes with controllable and efficient activity in biomedical diagnostics, drug delivery, and environmental chemistry.

## Introduction

Natural enzymes are exquisite biocatalysts that can catalyze almost every chemical transformation of life [1, 2]. However, there still exist inherent defects, such as poor stability and high cost. Since magnetite nanoparticles with intrinsic peroxidase-mimicking activity were firstly reported [3], the design and development of nanomaterials with enzyme-like activities (nanozymes) have attracted ever-growing research attention [4]. So far, a series of oxide- [5–7], metal- [8–10], and carbon-based nanomaterials [11–13] have been used to mimic horseradish peroxidase (HRP). Among these, noble metal nanomaterials [8, 14–19], such as silver (Ag) [15], gold (Au) [16, 17], platinum (Pt) [8], and palladium (Pd) [18, 19] et al., have been reported to possess high peroxidase-mimetic activities. On the other hand, introducing light to activate the activity of nanozymes has also been reported, which offers a wealth of opportunities for the biotechnology and chemical industry [4, 20]. However, present remote-controlled catalytic systems are still far from satisfactory.

Plasmonic metal nanomaterials can capture or respond to sunlight owing to the unique optical property of surface plasmon resonance (SPR). These metal nanomaterials, which can capture photons to generate hot electrons through SPR effect, have become the key materials for improving catalytic rate [21], promoting new optical sensing of biomolecules [22], engineering photothermal therapy, and using sunlight as renewable energy [23, 24]. However, none of them focused on the SPR effect of plasmonic metal-based nanozymes on their enzyme-mimicking activities. Thus, it would be attractive to combine plasmonic metal-based nanozymes with SPR effect to achieve highly active and light-tunable enzyme catalysis.

**Scheme 1:**
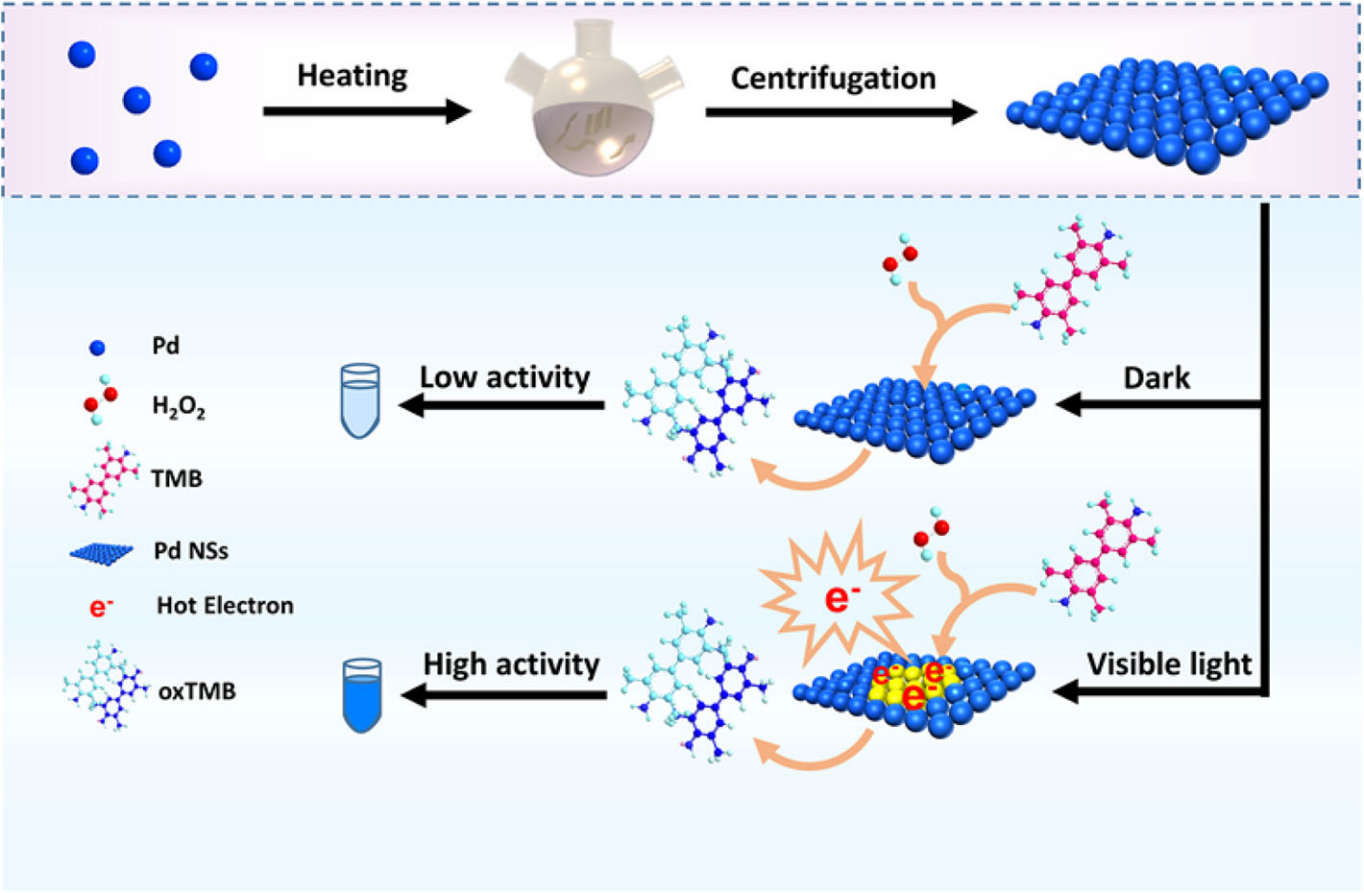
Schematic illustration of the Pd NSs structure and activity variation of Pd NSs under dark and visible light

Herein, we report for the first time that ultrathin Pd nanosheets (Pd NSs) with a thickness of about 1.1 nm can serve as an excellent and light-controllable peroxidase mimic. Pd-based nanozyme and light-controlled nanozyme systems have been reported to exhibit multiple enzyme mimetic activities including oxidase, peroxidase, catalase, and superoxide dismutase [4, 18, 19]. However, their structures are generally particles, bulks, and rods. As we all know, ultrathin Pd NSs have high surface energy, small lateral size, and high electron mobility, which results in the high density of active surface sites [25]. Besides, most of the atoms exposed to ultrathin nanosheets can be served as an ideal platform for engineering their performance [26]. Inspired by the unique structure of ultrathin nanosheets, we present a strategy that not only makes full use of the active sites of Pd atoms but also injects hot electrons into the enzyme catalysis process. As shown in Scheme 1, the interaction of photons with ultrathin Pd NSs can excite surface plasmon resonance that decays non-radiatively into hot electrons and holes, thus promoting the H_2_O_2_ in situ reductively decomposition to generate oxygen species radicals (O*) with oxidizing TMB under visible. By introducing visible light into the enzymatic reaction, the Pd NSs exhibit significantly higher catalytic activities than that of Pd NSs under dark conditions.

## Materials and Experimental

### Materials and Characterizations

The citric acid (CA); N, N-dimethylformamide (DMF); 3,3,5,5-tetramethylbenzidine (TMB); and cetyltrimethylammonium bromide (CTAB) were purchased from Sigma Aldrich. Pd (II) acetylacetonate, W(CO)_6_, and polyvinylpyrrolidone (PVP) were obtained from Macklin. Hydrogen peroxide (H_2_O_2_, 30 wt%) was bought from Beijing Chemicals (Beijing, China). All chemicals were used without further purification. Experimental water purified by Millipore system (18.2 MΩ; Millipore Co., USA) was used throughout the work.

The phase structures of the products were characterized by a PANalytical X-ray diffractometer that used Cu Kα radiation (*λ* = 1.5406 Å). And UV-vis diffuse reflectance spectra (DRS) of the samples were characterized at room temperature using the Lambda 750, PerkinElmer. The concentrations of catalysts in solutions were determined by the ICP-AES (720, Agilent). Transmission electron microscopy (TEM) images were recorded on JEM1400 plus transmission electron microscope operated at 100 kV. High-resolution TEM (HRTEM) analyses were performed on a JEM-2100F field emission transmission electron microscope (FE-TEM) at 200 kV.

### Synthesis of Pd Nanosheets

According to the reported method [27], Pd NSs were synthesized. Briefly, Pd (II) acetylacetonate (16 mg), PVP (MW = 24,000, 30 mg), CA (150 mg), and CTAB (60 mg) were mixed in DMF (10 mL) and stirred in a nitrogen atmosphere for 1 h. When the color of the solution in the flask (25 mL) changes into a uniform orange-red solution, 100 mg of W(CO)_6_ was added into the solution under an N_2_ atmosphere. Next, heat the flask at 80 °C for 1 h. After the reaction, the dark blue product was separated by centrifugation (9000 rpm/min, 3 min) with a sufficient amount of acetone and then dispersed in ethanol. This process was repeated three times. Finally, the Pd nanosheets were dispersed in 10 mL of ethanol for further experiments.

### Photocatalytic Peroxidase-Mimicking Measurement

The peroxidase-mimicking activity of the Pd NSs nanozymes was evaluated by measuring the oxidation of TMB. A 300-W Xe lamp (CEL-HXF300/CEL-HXUV300, China education Au-light Co., Ltd., Beijing) served as the light source, and adding a glass filter enables the visible light (*λ* ≥ 400 nm) to pass through. In a typical experiment, 12.6 μg mL^−1^ Pd NSs nanozymes (measured by ICP-AES) were added to 1 mL phosphoric acid buffer solution (0.1 M, pH 4) containing 50 mM H_2_O_2_ and 0.7 mM TMB at room temperature. During the different time under irradiation, the peroxidase-mimicking activity was determined by monitoring the characteristic absorption peak at 652 nm after centrifugal, which indicates the concentration of TMB oxidation products. The control experiment had the same conditions except for illumination.

## Results and Discussion

### Design and Characterization of the Pd NSs Nanozymes

**Fig. 1:**
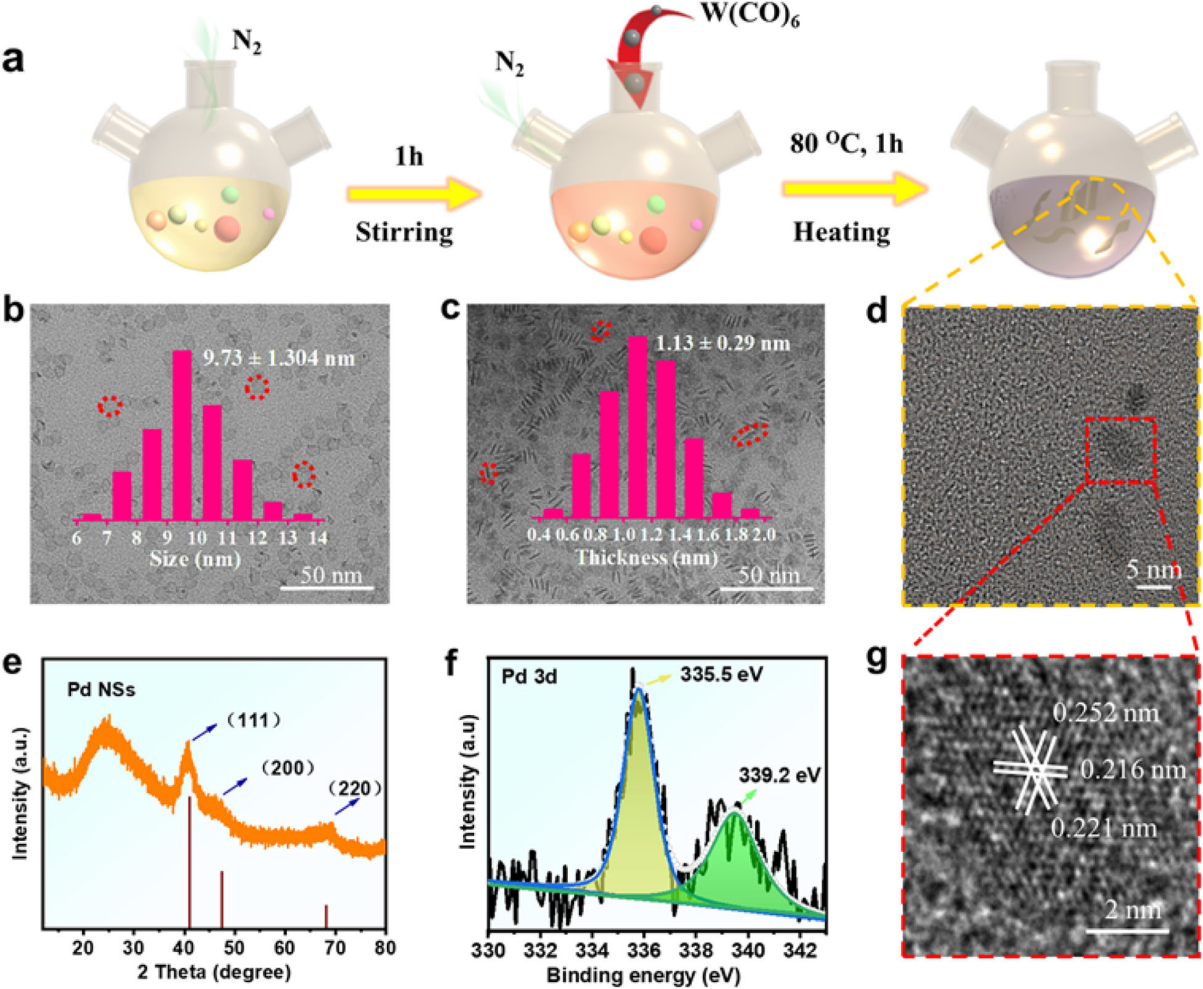
Characterization of the nanozymes. **a** Growth mechanism of Pd nanozymes. **b**, **c**, and **d** TEM. **e** XRD patterns. **f** Pd 3d XPS spectrum of Pd NSs. **g** HRTEM image of Pd NSs

A typical synthesis of palladium nanosheets was prepared through a classical method (Fig. 1a) to construct a series of highly active atomic sites ultrathin nanozymes that have inherent substrates and photons capturing and efficient peroxide enzyme-mimic characteristics. Figure 1 b–d show a typical low-magnification transmission electron microscopy (TEM) image of the synthesized Pd NSs, in which composed of uniform nanosheets with lateral dimensions of about 10.0 nm (Fig. 1b, inset) and the average thickness of about 1.1 nm (Fig. 1c, inset). According to the size, the area percentage of the top and bottom flat surface is over 90%. High-resolution transmission electron microscopy (HRTEM) is used to further confirm the morphology and phase of Pd NSs. Figure 1 g shows the appropriate spacings of ~ 0.22 and 0.256 nm for the (111) and the (200) lattice planes of palladium [27]. In order to meet the needs of the experiment, the XRD pattern of Pd NSs was characterized by loading on commercial carbon. As shown in Fig. 1e, the diffraction peaks at around 40.11, 46.65, and 68.12 correspond to (111), (200), and (220) planes of cubic Pd NSs, which is consistent with the observation from HRTEM. Moreover, no peaks indicative of crystalline phases other than the peaks associated with the commercial carbon. The Pd 3d spectrum exhibits two peaks (Fig. 1f) Pd 3d_5/2_ and Pd 3d_3/2_ (resulting from the spin-orbit splitting), located at respectively 335.5 and 339.2 eV [25], which revealed that there are two chemical environments for palladium atom.

### Photocatalytic Peroxidase-Mimicking Activity

**Fig. 2:**
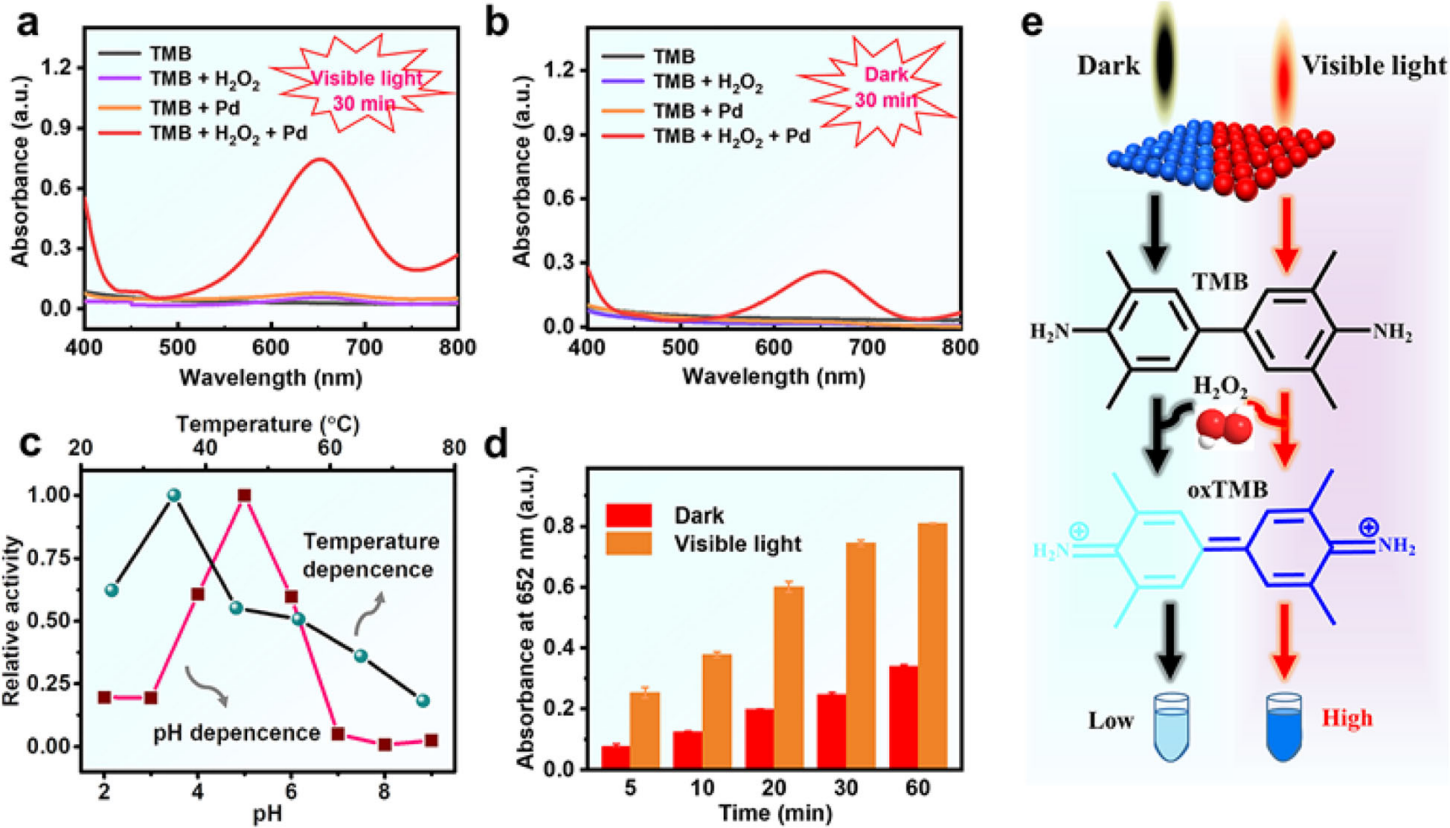
The peroxidase-mimicking activity of Pd NSs. **a-b** Typical UV-visible absorption spectra of different samples under visible light and dark conditions. **c** Temperature and pH effect on peroxidase-mimicking activity. **d** Time courses for peroxidase-mimicking activity. **e** The peroxidase-like mechanism of Pd NSs under dark and visible light. Experimental conditions: visible light = λ ≥ 400 nm, TMB = 0.7 mM, H_2_O_2_ = 50 mM, temperature =25 °C, Pd NSs = 12.6 μg/mL, and phosphate buffer solution (0.1 M, pH 4)

The peroxidase-like activity of the Pd NSs was investigated by using TMB as a typical peroxidase substrate. Since most of the Pd atoms were exposed on the surface of ultrathin nanosheets, we reason that ultrathin Pd NSs possess a high density of active surface sites and thus resulting in excellent catalytic activity. As expected, in the coexistence of H_2_O_2_, Pd NSs can efficiently catalyze the oxidation of colorless substrate TMB to blue product oxTMB, with the characteristic absorption at 652 nm (Fig. 2a, b). However, without the addition of H_2_O_2_, the activity of Pd NSs can be neglected under the same experimental condition, which revealed that the peroxidase-like activity played an important role during the reaction. Similar to natural enzymes and other nanozymes, Pd nanozymes have a pH-, temperature-, and concentration-dependent peroxidase activity (Fig. 2c and Fig. S1). Under the experiment condition, Pd NSs showed optimized catalytic activity in the weak acid solution, and the characteristic absorbance peak of the reaction solution was the highest at 35 °C when the temperature varied from 25 to 75 °C (Fig. 2c). Surprisingly, with or without light irradiation, a significant difference in peroxidase-mimicking activity was observed (Fig. 2d and Fig. S2). According to the absorption value of the reaction solution for 60 min, the activity of Pd NSs under visible light exhibited approximately 2.4~3.2 times higher than that of Pd NSs under dark conditions (Fig. 2d and Fig. S2). Similarly, the introduction of light into the catalysis process other plasmonic metal nanoparticles can also increase their peroxidase-like activity (Fig. S3–S5). By comparison of these nanozymes, we found that Pd NSs showed the greatest range of activity regulation. Such a phenomenon is mainly due to the unique structure of the ultrathin nanosheet. From these obtained results, we can infer that visible light has a direct effect on the peroxidase-like activity of all plasmonic metal nanomaterials (Fig. 2e), and SPR effect may play an essential role in the catalytic process.

### Kinetic and Mechanism Investigation of Pd nanozymes

**Fig. 3:**
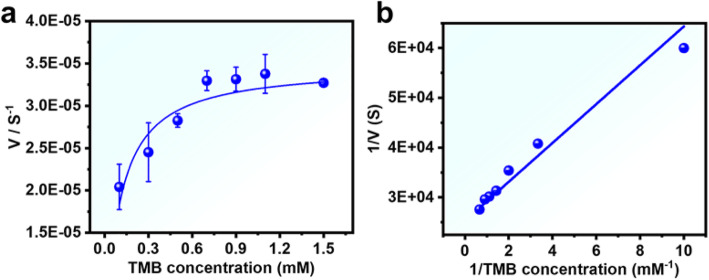
Steady-state kinetic assay and catalytic mechanism of Pd NSs (12.6 μg/mL). **a** The concentration of H_2_O_2_ was 50 mM, and the TMB concentration was varied (0.1–1.5 mM). **b** Double reciprocal plots for TMB

To characterize the enzymatic behavior of Pd NSs, we determined the enzyme kinetics theory for the reaction. However, within the suitable concentration range of TMB, Pd NSs present a typical Michaelis-Menten curve (Fig. 3a). The Michaelis constant (Km) and the maximum reaction speed (Vmax) were obtained by using the Lineweaver Burk equation, as shown in Table S1. Compared with horseradish peroxidase (HRP), the apparent Km value of the Pd NSs with TMB was weakened by 0.28 (Fig. 3a, b and Table S1). This result indicates that the ultrathin sheet structure of as-prepared Pd NSs exhibits high affinity to TMB, even higher than that of natural enzyme HRP.

**Fig. 4:**
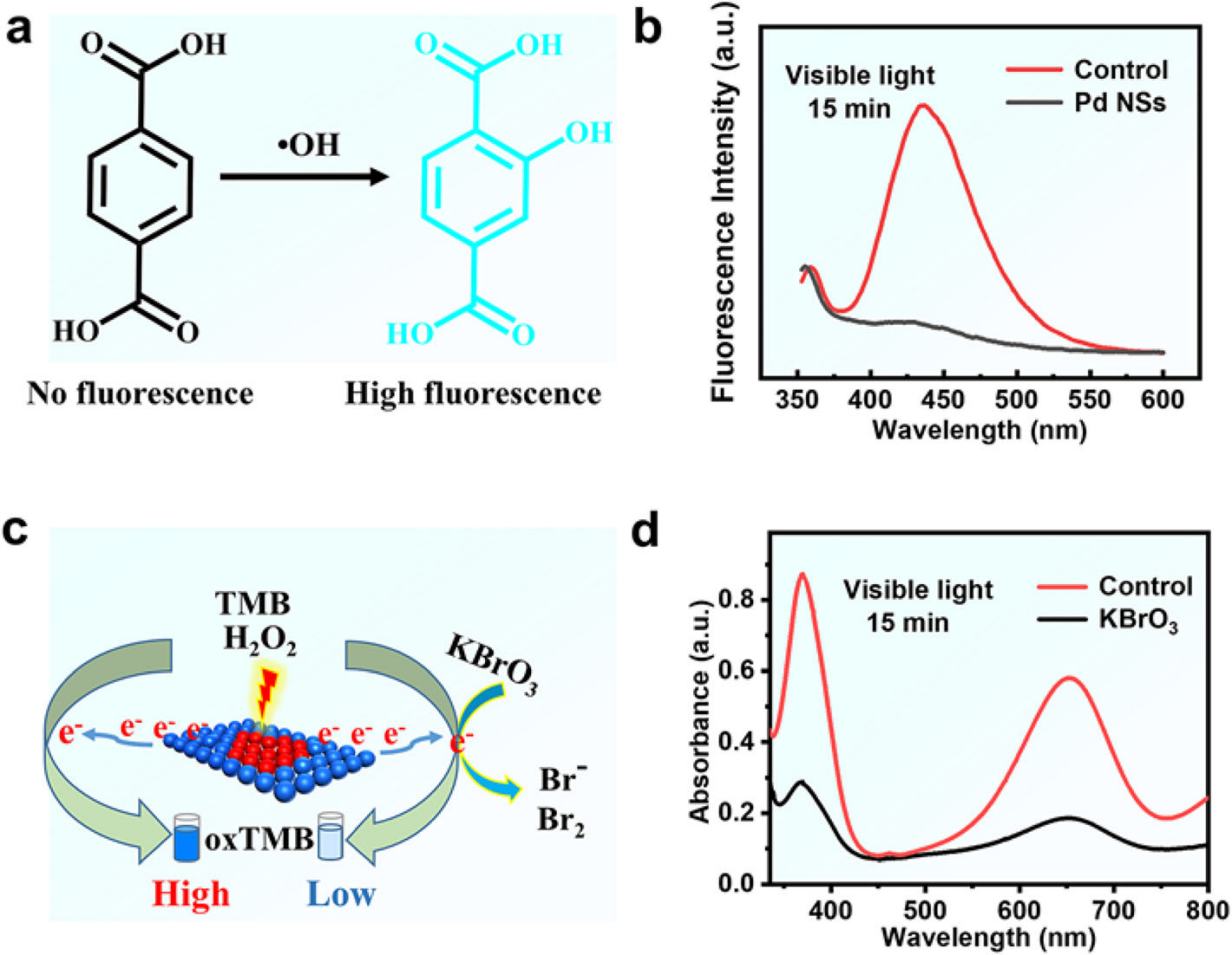
**a** Mechanism diagram of terephthalic acid (TA) capturing hydroxyl radicals (•OH). Spectra of samples containing phosphate buffer (0.1 M, pH 4), H_2_O_2_ (50 mM), and visible light illumination (*λ* ≥ 400 nm, 15 min). **b** The fluorescence emission spectra in the presence of Pd NSs (12.6 μg/mL) and TA (66.7 μM). **c** Mechanism diagram of KBrO_3_ capturing hot electronics. **d** The absorption spectrum in the presence of Pd NSs (12.6 μg/mL), KBrO_3_ (0.3 mg/mL), and TMB (0.7 mM)

Since it is clear that H_2_O_2_ can be decomposed to form reactive oxygen species with Pd NSs, it is crucial to understand what species are produced to provide an oxidation function. In principle, noble metals can catalyze the decomposition of H_2_O_2_ to form •OH, and reaction intermediates O* at lower pH conditions [28], either of which may be the species that provide oxidation function in enzyme-mimetic reactions. In order to understand the possible catalytic mechanism of Pd NSs, we firstly used terephthalic acid (TA)/H_2_O_2_ system to test whether the peroxidase-like characteristics of Pd NSs are related to the formation of •OH radicals (Fig. 4a). Using TA as a fluorescent probe, a highly fluorescent product was produced by the reaction of 2-hydroxyterephthalic acid with •OH [29]. As shown in Fig. 4b, the fluorescence intensity of the solution decreases significantly after Pd NSs addition. The results are in good consistent with the fluorescence intensity decreased with the increase of Pd NSs concentration (Fig. S6). These results indicate that Pd NSs can consume •OH radicals rather than generate them. Hence, similar to the reported catalytic behavior of ferritin-platinum nanoparticles [30], the catalytic performance of our Pd NSs was independent of the formation of •OH radical.

To investigate whether the catalytic mechanism of Pd NSs relates to the formation of hot electrons by light, we also explore the trapping experiment of active species hot electrons during the photocatalytic reaction (Fig. 4c) [31]. As can be seen from Fig. 4d, the catalytic ability of Pd NSs toward TMB oxidation decreases significantly within 15 min by the addition of 0.3 mg/mL KBrO_3_ (a quencher of e^−^). Such a huge difference between the KBrO_3_/reaction system and pure system reveals that the presence of hot electrons might be critical for TMB oxidation. This is in accordance with the results of Fig. S7 that Pd NSs have a broad absorption peak through SPR effect in the spectral range of 500–1000 nm [25]. Besides, once hot electrons moved away from the surface of Pd NSs, there are corresponding holes left on their surface. Since these holes can oxidize ethanol to produce acetaldehyde, they might also have powerful oxidization ability toward TMB. As expected, without the addition of H_2_O_2_, more oxTMB was created under the illumination of visible light.

**Fig. 5:**
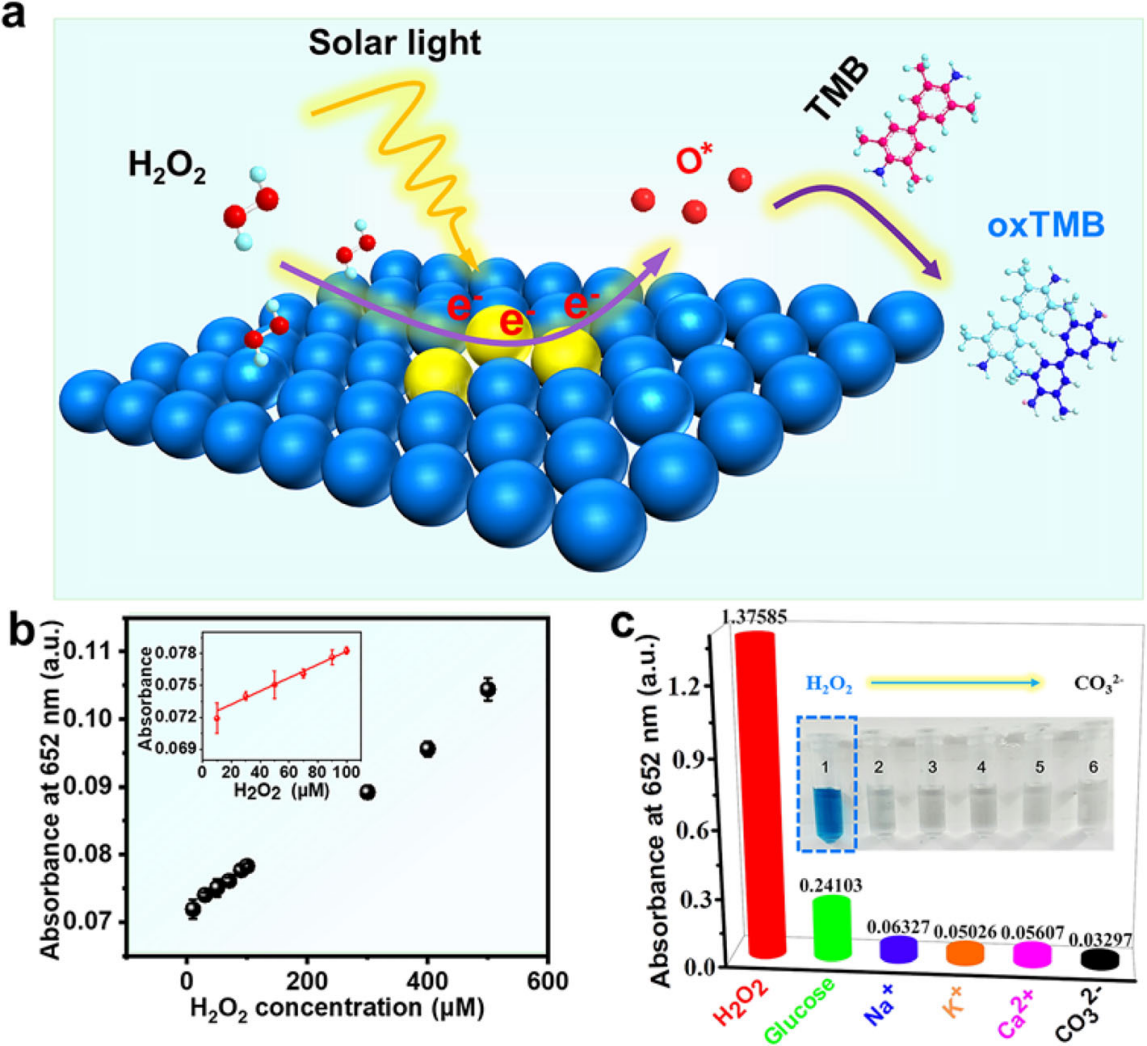
**a** Schematic diagram of a sensor for H_2_O_2_ detection. **b** Dose-response curve of different hydrogen peroxide concentration. Experimental conditions: Pd nanozyme (25.2 μg/mL), phosphate buffer (0.1 M, pH 4), and TMB (0.7 mM) visible light illumination (*λ* ≥ 400 nm, 3 min). Inset: linear calibration plots. **c** Interference of other impurities on the absorbance of H_2_O_2_ colorimetric sensor at 652 nm. Experimental conditions: Pd nanozyme (25.2 μg/mL), phosphate buffer (0.1 M, pH 4), TMB (0.7 mM), visible light illumination (*λ* ≥ 400 nm, 15 min), and including 50 mM H_2_O_2_, 200 mM glucose, Na^+^, K^+^, Ca^*2*+^, and CO_3_^2−^, and the inset shows the color changes of reaction solutions

Next, we test whether reactive oxygen species were formed by the activation of O_2_ under visible light, including superoxide (O_2_^−^). In light of this, controlled experiments were performed under different atmospheres. For Fig. S8, the catalytic performance of mimetic enzymes does not significantly change when we are introduced nitrogen and oxygen with saturating the reaction system, respectively, which is not considerably affected by the O_2_ for the photocatalytic activity of Pd NSs. It is essential to point out that the ultimate performance of Pd NSs, even up to 0.051 a.u./min for 5 min under visible light, was 3.2 times higher than that of the Pd NSs catalysts in the dark (Fig. 2d). The extremely high activity of Pd nanozyme under visible light leads to a hypothesis that the existence of hot electrons by the SPR effect of Pd NSs promoting the formation of reaction intermediates O* instead of free radical account for a peroxidase-like activity (Fig. 5a) [28]. In brief, the trapping experiment of active species and ventilation experiment provide sturdy support for the photocatalytic mimetic enzyme mechanism of Pd NSs.

### A New Real-Time and Highly Sensitive Sensor

This was demonstrated in several studies that the introduction of light into the sensor as an input of external energy can improve the performance of the sensor [22, 32, 33]. For example, Ling et al. [32] found that O_2_ sensing properties of the 10 at. % LaOCl-SnO_2_ sensor was significantly improved by ultraviolet light illumination. Considering the significant influence of light on the sensor and the excellent peroxidase-mimicking activity of Pd NSs under visible light in our experiments, an effective and sensitive colorimetric sensor H_2_O_2_ was built. The mechanism of the sensor (Fig. 5a) shows Pd NSs can make full use of its large specific surface area to capture photons and generate a large number of hot electrons. After that, the hot electron promotes the decomposition of H_2_O_2_ to produce reaction intermediates O*, which can oxidize TMB to blue oxTMB. Finally, the efficient detection of H_2_O_2_ was realized.

As can be seen from the inset of Fig. 5b, the linear range of the constructed H_2_O_2_ sensor by us was from 10 to 100 μM, and the calculation of detection limit was 13.40 μM (LOD = 3 *s*/*k*, where *s* and *k* represent the linear calibration blocks of the relative standard deviation and slope of eight parallel control measurements, respectively. In this work, *s* = 2.97988 × 10^−4^, *k* = 6.67 × 10^−5^). Therefore, hydrogen peroxide sensor based on Pd NSs was superior to other reported nanomaterials under the condition of introducing light. From Table S2, it can be seen that with the same colorimetric method to detect hydrogen peroxide, and our sensor shows a broad range of linearity [34]. And the detection limit was lower than many sensors based on Fe-based or Co-based peroxidase mimics (Table S3) [35, 36]. Finally, we carried out H_2_O_2_ and a series of control experiments (Fig. 5c) with potential interferences such as K^+^, glucose, Na^+^, CO_3_^2−^, and Ca^2+^. As shown in the inset of Fig. 5c, it is obvious that the absorbance of these interferences is weak at 652 nm, and the color does not change. Based on our results, an efficient and highly specific hydrogen peroxide sensor based on visible light has been successfully realized. This sensor not only makes full use of visible light to improve its detection performance but also provides a good example for other plasmonic metals in the sensor.

## Conclusions

In summary, we demonstrated an exciting example of applying ultrathin Pd nanosheets (Pd NSs) as a highly efficient and light-controllable peroxidase mimic, owing to a high density of active sites on the surface of nanosheets and unique optical property of SPR. With the irradiation of visible light, the generated hot electrons from Pd nanosheets via SPR effect can subsequent decompose H_2_O_2_ to produce intermediates O*. Under visible light irradiation, such nanozymes exhibited much higher peroxidase-like activity than that of in the dark. Such a light-activated system was further used for the enhanced biosensing of H_2_O_2_. The basic concept presented here, based on the generation of hot electrics through SPR effect on the photoactivated Pd nanozymes, might contribute to the design of smart or more efficient artificial enzyme systems and offer many new opportunities for the chemical industry and biotechnology.

## Supplementary information

**Additional file 1: **Synthesis of Au, Ag, and Cu NPs. **Fig. S1** TMB effect on peroxidase-mimicking activity. **Fig. S2** Absorption spectrum changes of the reaction solution. (a) Visible light, (b), and (c) Dark. **Fig. S3-S5** (a) One-step formation of Au, Ag, and Cu NPs, (b) The UV−vis absorption spectra, (c) The peroxidase mimicking in the assayed reaction system, (d) Time courses for peroxidase-like activity, respectively. **Fig. S6** The fluorescence emission spectra in the presence of different concentration Pd NSs. **Fig.S7** The UV−vis absorption spectra of Pd NSs. **Fig. S8** Experiments in different atmospheres. **Table S1** Comparison of the Kinetic Parameters of Pd NSs and HRP. **Table S2** Comparison of the linear range of H_2_O_2_ by means of different sensors. **Table S3** Comparison of the limit of detection of H_2_O_2_ by means of different sensors.

## Data Availability

All data generated or analyzed during this study are included in this published article.

## Abbreviations

Pd NSs: Pd nanosheets
Ag: Silver
Au: Gold
Pt: Platinum
Pd: Palladium
SPR: Surface plasmon resonance
TEM: Transmission electron microscopy
HRTEM: High-resolution TEM
XRD: X-ray diffraction
HRP: Horseradish peroxidase
Km: Michaelis constant
O_2_^−^: Superoxide
