# Supplementary material for: Hot-Electron-Activated Peroxidase-Mimicking Activity of Ultrathin Pd Nanozymes
Source: Nanoscale Res Lett. 2020 Aug 11;15:162. doi: 10.1186/s11671-020-03388-9 (PMC7419420; doi:10.1186/s11671-020-03388-9)
Supplement: Supplementary file 1 — Additional file 1: Synthesis of Au, Ag, and Cu NPs. Fig. S1 TMB effect on peroxidase-mimicking activity. Fig. S2 Absorption spectrum changes of the reaction solution. (a) Visible light, (b), and (c) Dark. Fig. S3-S5 (a) One-step formation of Au, Ag, and Cu NPs, (b) The UV−vis absorption spectra, (c) The peroxidase mimicking in the assayed reaction system, (d) Time courses for peroxidase-like activity, respectively. Fig. S6 The fluorescence emission spectra in the presence of different concentration Pd NSs. Fig.S7 The UV−vis absorption spectra of Pd NSs. Fig. S8 Experiments in different atmospheres. Table S1 Comparison of the Kinetic Parameters of Pd NSs and HRP. Table S2 Comparison of the linear range of H2O2 by means of different sensors. Table S3 Comparison of the limit of detection of H2O2 by means of different sensors. [file 11671_2020_3388_MOESM1_ESM.doc]

| **Supplementary information**  Hot-electron-activated peroxidase-mimicking activity of ultrathin Pd nanozymes |
| --- |

Yonghua Tang^1⊥^, Xueqing Xiong^1⊥^, Chengjie Xu^1^, Deshuai Yu^1^, Yanyan Huang^2^, Changxu Lin^1*^, Xiangyang Liu^1,3^, and Youhui Lin^1*^

^1^Research Institute for Biomimetics and Soft Matter, Department of Physics, Fujian Provincial Key Laboratory for Soft Functional Materials Research, Jiujiang Research Institute, Xiamen University, Xiamen 361005, China.

^2^College of Light Industry and Food Engineering, Nanjing Forestry University, Nanjing 210037, China.

^3^Department of Physics, National University of Singapore, 2 Science Drive 3, Singapore, 117542, Singapore.

* Correspondence: linyouhui@xmu.edu.cn.

**Synthesis of Au, Ag, and Cu NPs**

The metal nanoparticles were prepared through the reduction of HAuCl_4_, AgNO_3_, and CuCl_2_ by NaBH_4_. Briefly, 10 µL of 0.1 M NaBH_4_ was added to 1 mL of an aqueous solution containing 50 mM HAuCl_4_, AgNO_3_, or CuCl_2_ for 2 min. Then the products were washed twice with water and dispersed in 1 mL of water for the later experiment.

**Figure S1** TMB effect on peroxidase-mimicking activity. Experimental conditions : H_2_O_2_ = 50 mM, Temperature = 25 °C, Pd NSs = 12.6 mg/mL and phosphate buffer solution (0.1 M, pH 4).

**
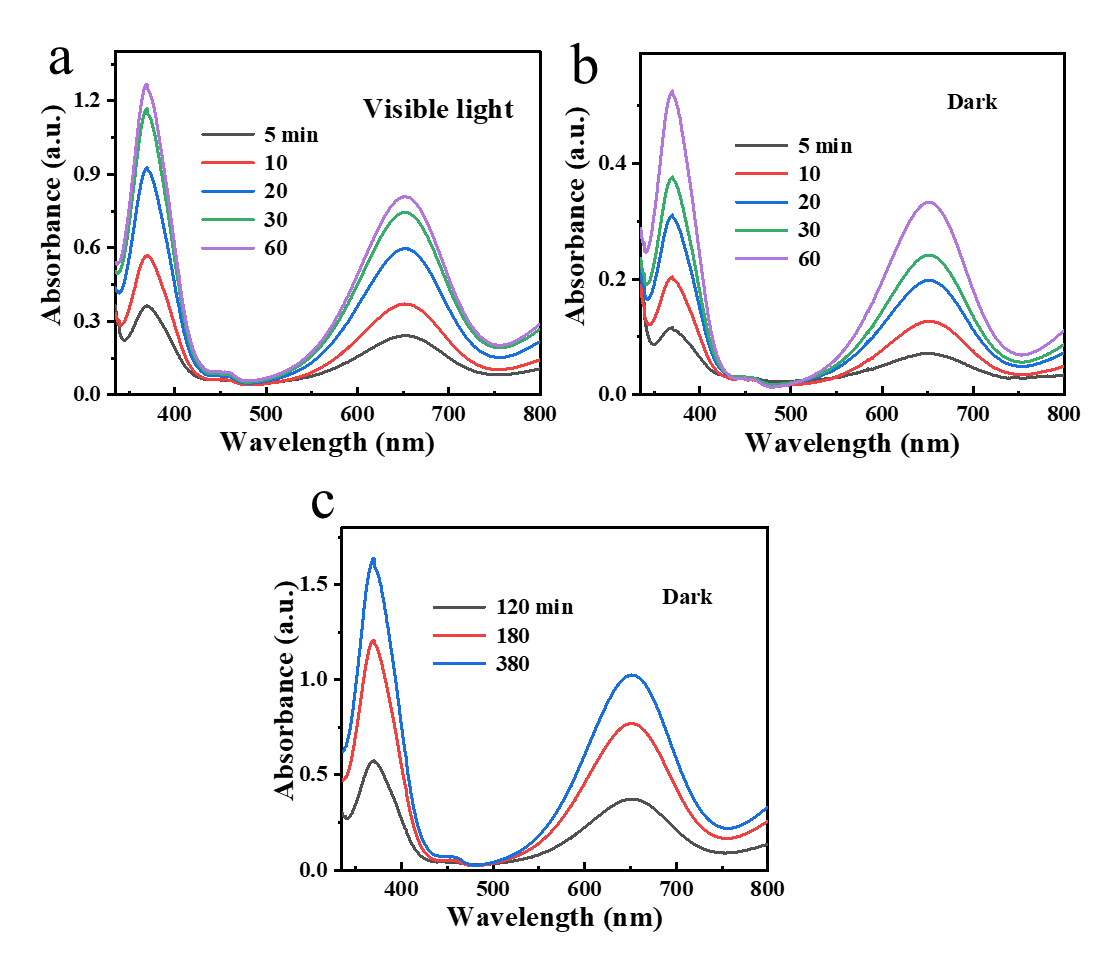
**

**Figure S2** Absorption spectrum changes of the reaction solution. (a) Visible light, (b) and (c) Dark.


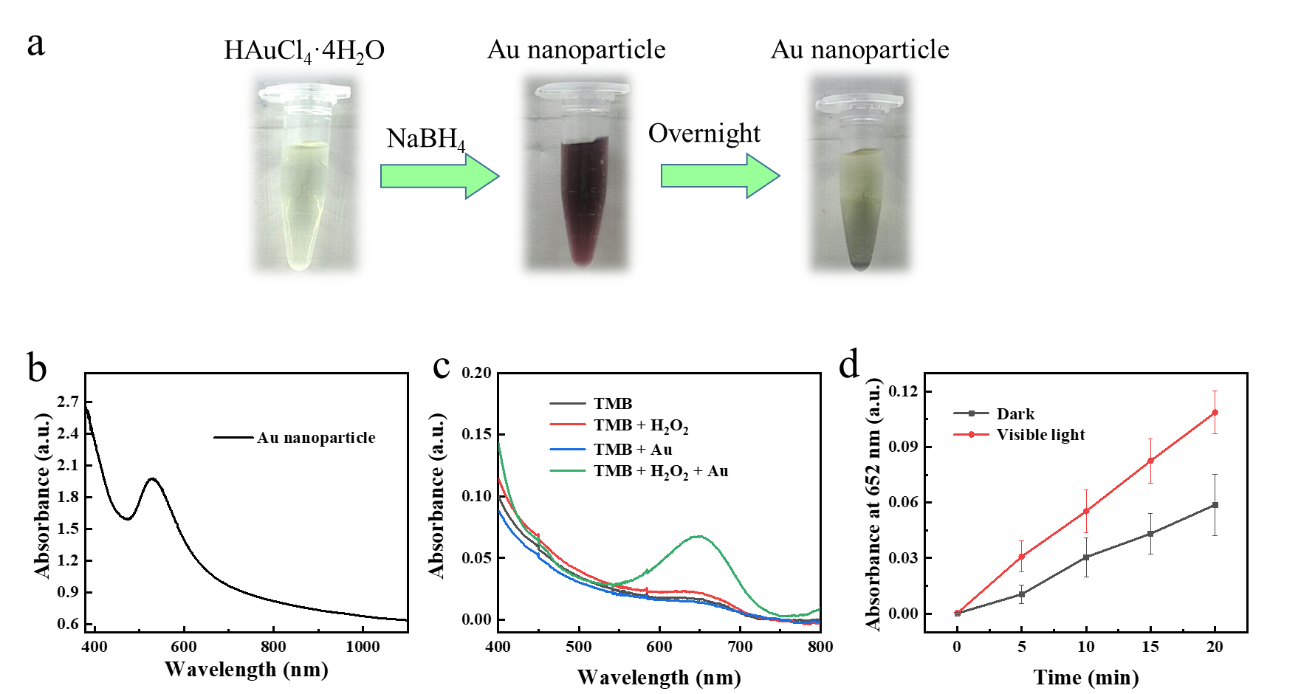


**Figure S3** (a) One-step formation of Au NPs. (b) The UV−vis absorption spectra. (c) The peroxidase mimicking in the assayed reaction system. (d) Time courses for peroxidase-like activity.


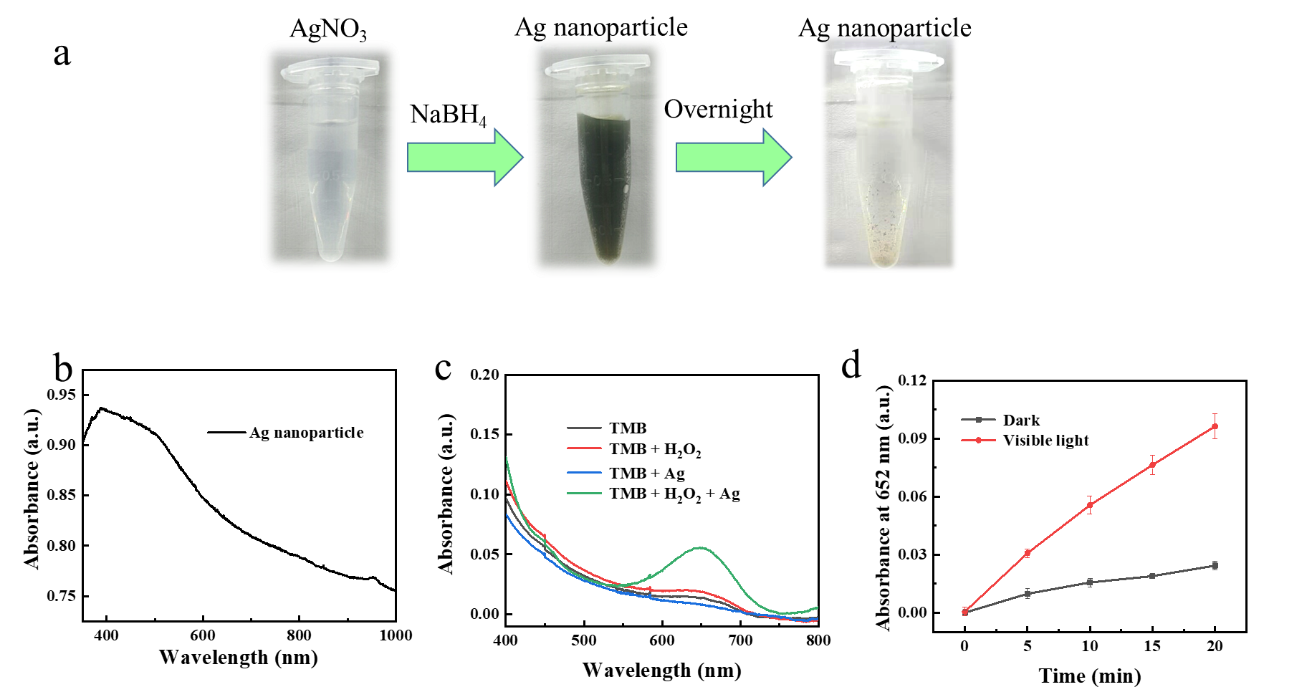


**Figure S4** (a) One-step formation of Ag NPs. (b) The UV−vis absorption spectra. (c) The peroxidase mimicking in the assayed reaction system. (d) Time courses for peroxidase-like activity.


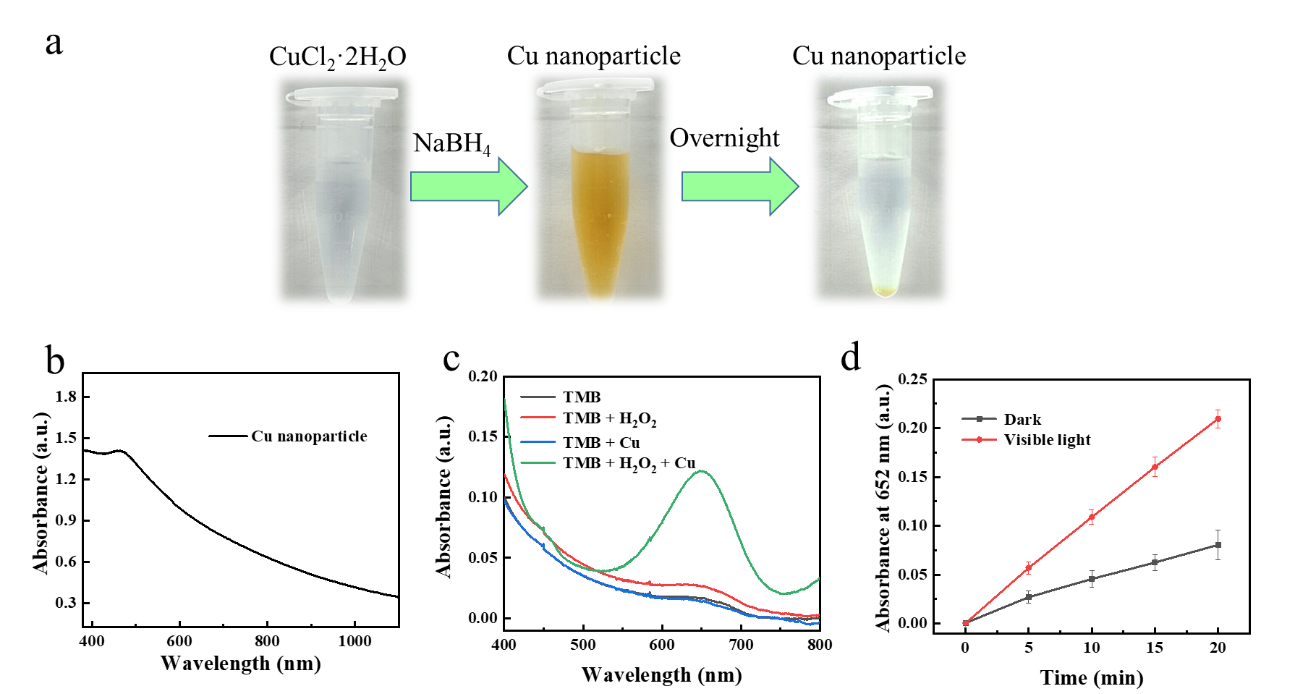


**Figure S5** (a) One-step formation of Cu NPs. (b) The UV−vis absorption spectrum. (c) The peroxidase mimicking in the assayed reaction system. (d) Time courses for peroxidase-like activity.


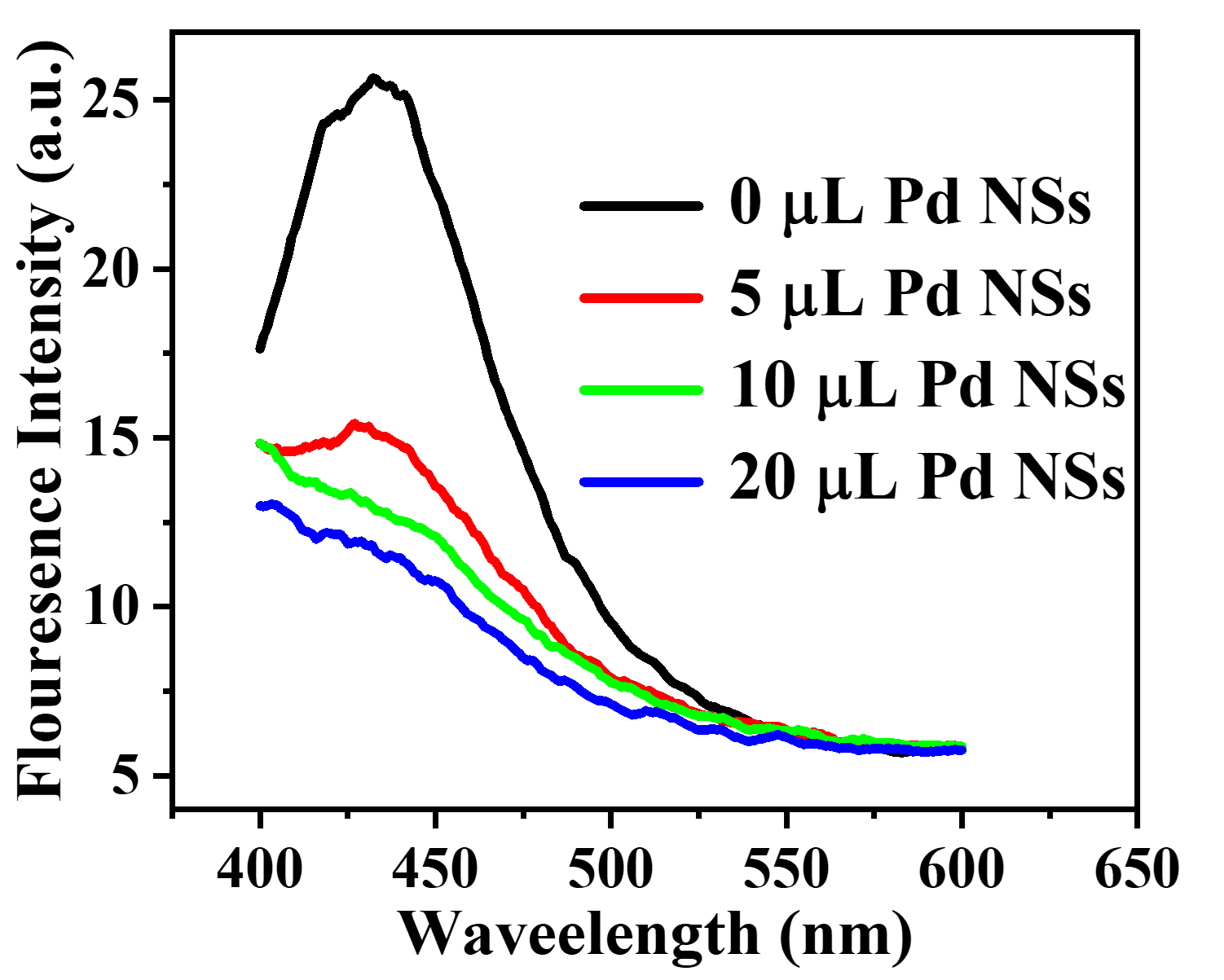


**Figure S6** The fluorescence emission spectra in the presence of different concentration Pd NSs. Spectra of samples containing phosphate buffer (1 mL, 0.1 M, pH 4), H_2_O_2_ (50 μL, 1 M), TA (50 μL, 4 mM), and visible light illumination (λ≥400 nm, 15 min).

**Figure S7** The UV−vis absorption spectra of Pd NSs.


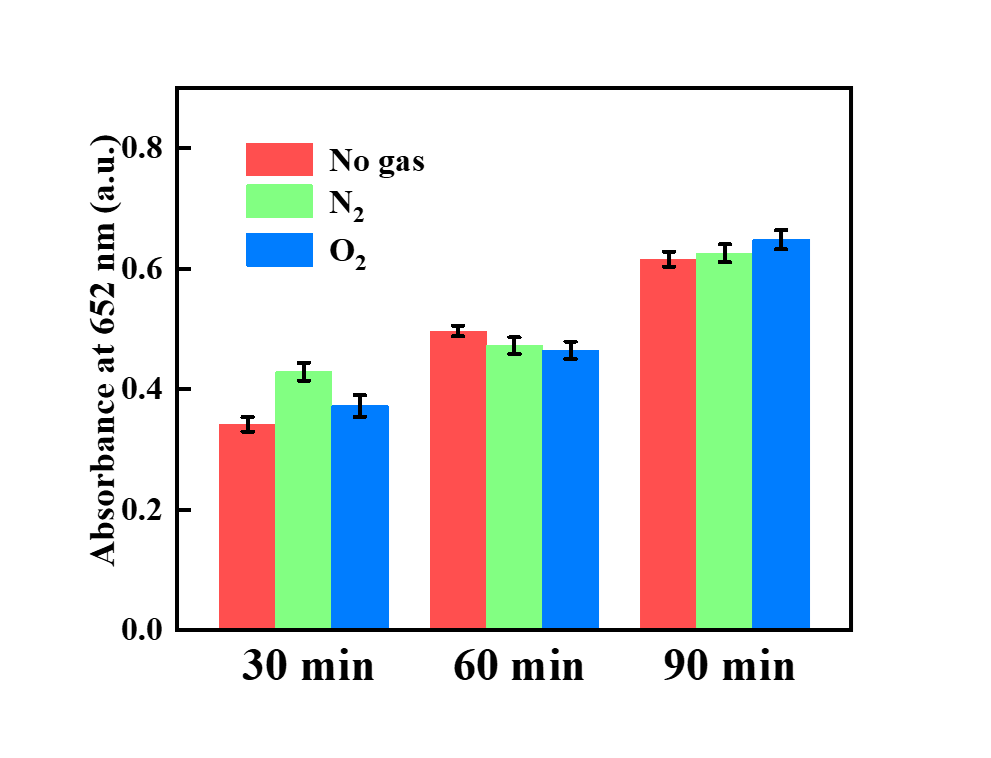


**Figure S8** Experiments in different atmospheres. [Visible light irradiation] = λ ≥ 400 nm, [TMB] = 1 mM, [H_2_O_2_] =2. 5 mM, [Pd NSs] = 25.2 mg/mL and phosphate buffer solution (0.1 M, pH 4).

**Table S1.** Comparison of the Kinetic Parameters of Pd NSs and HRP.


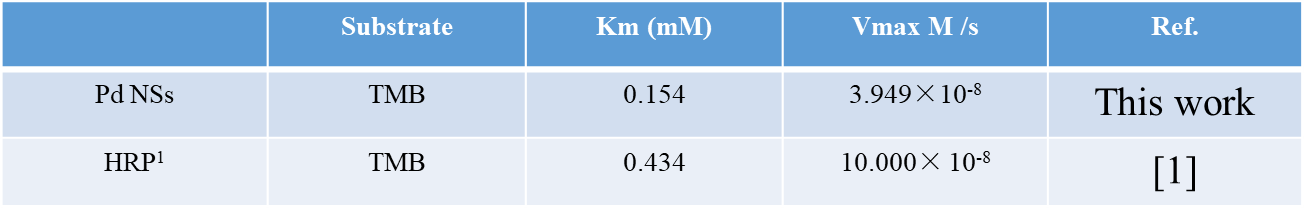


**Table S2.** Comparison of the linear range of H_2_O_2_ by means of different sensors.


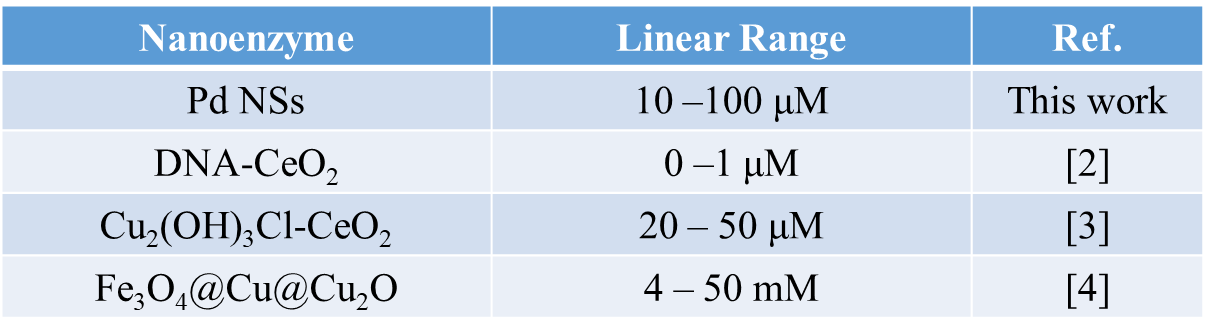


**Table S3.** Comparison of the limit of detection of H_2_O_2_ by means of different sensors.


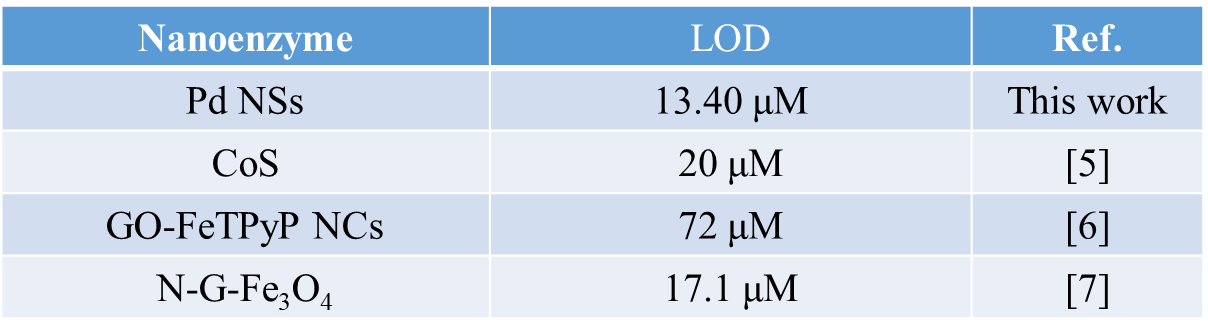


**Reference**

[1] Gao, L. Z.; Zhang, J.; Nie, L.; Zhang, J. B.; Zhang, Y.; Gu, N.; Wang, T. H.; Feng, J. D.; Yang, L.; Perrett, S.; Yan, X. Y. Intrinsic peroxidase-like activity of ferromagnetic nanoparticles. *Nat. Nanotechnol.*, **2007**, *2*, 577-583.

[2] Liu, B.; Sun, Z. Y.; Huang, P. J.; Liu, J. W. Hydrogen Peroxide Displacing DNA from Nanoceria: Mechanism and Detection of Glucose in Serum. *J. Am. Chem. Soc.*, **2015**, *137*, 1290-1295.

[3] Wang, N.; Sun, J. C.; Chen, L. J.; Fan, H.; Ai, S. Y. A Cu_2_(OH)_3_Cl-CeO_2_ nanocomposite with peroxidase-like activity, and its application to the determination of hydrogen peroxide, glucose and cholesterol. *Microchim. Acta*, **2015**, *182*, 1733-1738.

[4] Wang, Z. H.; Chen, M.; Shu, J. X.; Li, Y. One-step Solvothermal Synthesis of Fe_3_O_4_@Cu@Cu_2_O Nanocomposite as Magnetically Recyclable Mimetic Peroxidase. *J. Alloys. Compd.*, **2016**, *682*, 432-440.

[5] Yang, H. G.; Zha, J. Q.; Zhang, P.; Xiong, Y. H.; Su, L. J.; Ye, F. G. Sphere-like CoS with nanostructures as peroxidase mimics for colorimetric determination of H_2_O_2_ and mercury ions. *RSC. Adv.*, **2016**, *6*, 66963-66970.

[6] Socaci, C.; Pogacean, F.; Biris, A. R.; Coros, M. M.; Rosu, C.; Magerusan, L.; Katona, G.; Pruneanu, S. Graphene oxide vs. reduced graphene oxide as carbon support in porphyrin peroxidase biomimetic nanomaterials. *Talanta*, **2016**, *148*, 511-517.

[7] Zhang, W. J.; Chen, C. P.; Yang, D. X.; Dong, G. X.; Jia, S. J.; Zhao, B. X.; Yan, L.; Yao, Q. Q.; Sunna, A.; Liu, Y. Optical Biosensors Based on Nitrogen‐Doped Graphene Functionalized with Magnetic Nanoparticles. *Adv. Mater. Interfaces.*, **2016**, *3*, 1600590.
